# Characterization of Fruit Quality Attributes and Cell Wall Metabolism in 1-Methylcyclopropene (1-MCP)-Treated ‘Summer King’ and ‘Green Ball’ Apples During Cold Storage

**DOI:** 10.3389/fpls.2019.01513

**Published:** 2019-11-21

**Authors:** Nay Myo Win, Jingi Yoo, Soon-Il Kwon, Christopher B. Watkins, In-Kyu Kang

**Affiliations:** ^1^Department of Horticultural Science, Kyungpook National University, Daegu, South Korea; ^2^Apple Research Institute, National Institute of Horticultural and Herbal Science, RDA, Gunwi, South Korea; ^3^School of Integrative Plant Science, Horticulture Section, Cornell University, Ithaca, NY, United States

**Keywords:** cell wall component, cell wall-modifying enzyme, Malus domestica Borkh, relative gene expression, softening

## Abstract

This study aimed to elucidate whether 1-methylcyclopropene (1-MCP) treatment delays the fruit softening mechanism associated with the fruit quality of the newly released apple cultivars “Summer King” and “Green Ball” during cold storage. For both cultivars, the fruit treated with 1-MCP exhibited lower internal ethylene concentration, higher firmness, and higher titratable acidity relative to the control fruit, in association with less fruit softening. In addition, the treated fruit significantly delayed fresh weight loss and reduction of soluble solids content, especially in “Green Ball.” Moreover, slower degradation of cell wall components (water-soluble pectin, sodium carbonate-soluble pectin, hemicellulose, and cellulose) was also observed in the treated fruit in comparison to the control fruit. Similarly, the enzymatic activities (of polygalacturonase, pectin methylesterase, cellulase, β-galactosidase, and α-L-arabinofuranosidase) that cause cell wall degradation were relatively lower in the treated fruit than in the control fruit for both cultivars, which was further proved by transcriptional analysis of the genes encoding the enzymes. Overall, the results suggested that the usage of 1-MCP is useful to delay fruit softening of the two cultivars during cold storage by delaying the degradation of cell wall components and enzymatic activities of cell wall hydrolysis.

## Introduction

The apple industry is relatively large among the other fruit industries in Korea. However, fruit softening that negatively affects marketable quality traits, such as crisp texture, flavor, and appearance, impacting consumers’ preference continue to be a problem for the industry. Fruit softening negatively affects other fruit quality factors too, such as the soluble solids contents (SSC) and titratable acidity (TA), which positively influence consumer acceptability and palatability (Harker et al., 2008; Oritz et al., 2011a). Hence, fruit softening is a major problem in the marketplaces and causes economic loss to the growers. Fruit softening is generally a result of changes to the cell wall components (such as pectin, hemicellulose, and cellulose), which are associated with activities of cell wall-degrading enzymes, such as polygalacturonase (PG), pectin methylesterase (PME), cellulase, β-galactosidase (β-GAL), and α--arabinofuranosidase (α-ARF) (Deng et al., 2005; Wei et al., 2010; Wang et al., 2015; Gwanpua et al., 2016; Chen et al., 2017a; Chen et al., 2017b; Lu et al., 2018; Yoo et al., 2018).

To delay the fruit softening mechanism, 1-methylcyclopropene (1-MCP) has been increasingly exploited in the storage of fruits, retaining the fruit quality attributes by slowing the decrease in firmness and TA, which are directly linked to delaying of the fruit softening mechanism during postharvest storage (Blankenship and Dole, 2003; Mattheis, 2008; Yoo et al., 2013). The benefit of using 1-MCP to delay fruit softening in apples had also been well documented (Watkins, 2006; Watkins, 2008; Kim et al., 2018). In apples, 1-MCP delayed fruit softening during storage by inhibiting ethylene production and activities of cell wall-degrading enzymes (Watkins, 2006; Lu et al., 2018; Yoo et al., 2018). Recently, Kim et al. (2018) observed a reduction in the loss of firmness, TA, SSC, and water, accompanied by less fruit softening, during cold storage of 1-MCP-treated ‘Gamhong’ apple.

Furthermore, the involvement of the genes (*β-GAL*, *α-ARF*, *PG1*, and *PME1*) responsible for regulating the corresponding activities of the cell wall-degrading enzymes during storage has also been revealed (Wei et al., 2010; Gwanpua et al., 2016). However, Gwanpua et al. (2016) found no variation in pectin compounds between the cultivars ‘Jonagold’ and ‘Granny Smith’ treated with 1-MCP, while Lu et al. (2018) described a negative effect of 1-MCP on aroma quality of ‘Fuji’ apples. Therefore, it was likely that the role of 1-MCP in delaying of fruit softening and maintenance of other fruit quality attributes is highly dependent on the cultivar, fruit maturity, and storage condition (Rupasinghe et al., 2000; Watkins et al., 2000; Delong et al., 2004).

The two cultivars ‘Summer King’ and ‘Green Ball’, which were recently developed by the Apple Research Institute (NIHHS, RDA) in Gunwi, Korea, are increasingly popular in the Korean apple industry. However, a year-round supply of quality apples to the markets has not been possible yet, due to lack of an appropriate storage method. Therefore, it is essential to develop a storage method that maintains fruit quality for an extended time. In this context, the present study was conducted to investigate whether exploitation of 1-MCP can extend the storability of ‘Summer King’ and ‘Green Ball’, without fruit softening. In addition, the factors determining the fruit quality attributes during cold storage were identified. Furthermore, the mechanism by which 1-MCP delayed fruit softening was also investigated by analyzing the cell wall components and activities of cell wall-degrading enzymes, and the expression levels of the genes encoding these enzymes.

## Materials and Methods

### Plant Materials and Treatment

‘Summer King/M.9-T337’ (‘Fuji’ × ‘Golden Delicious’) and ‘Green Ball/M.9-T337’ (‘Golden Delicious’ × ‘Fuji’) apples were harvested on August 2 and 28, 2017, respectively, from three 6-year-old trees growing at the Mungyeong Apple Research Center (36°35′10.133″N, 128°11′12.469″ E), Gyeongsanbuk-do, the Republic of Korea. All fruit were carefully transported to the laboratory of Horticultural Crops Quality, Kyungpook National University, Daegu, the Republic of Korea. Fruit of uniform size (230–250 g for ‘Summer King’ and 280–300 g for ‘Green Ball’), without defects, were selected for the study. A total of 165 fruit were harvested, with 75 fruit each for the control and 1-MCP treatment.

A concentration of 1 µl L^−1^ 1-MCP (SmartFresh™, 3.3% active ingredient; AgroFresh Co., Yakima, WA, USA) was applied to the 75 fruit for 18 h in an enclosed 135-L container (Watkins et al., 2000). Untreated and 1-MCP-treated fruit were then stored together at 0°C, 90–95% relative humidity for up to 5 months of cold storage. Fifteen fruit of each treated and untreated apple cultivar were removed at 1-month intervals and assessed after 12 h at 20°C.

### Assessment of Fruit Quality Attributes

The flesh firmness was measured using a rheometer (Compac-100II, Sun Scientific Co., Tokyo, Japan) on 15 individual fruit at three locations in the equator regions of each apple. The juice expressed from each fruit was used to determine the SSC and TA. SSC was measured using a refractometer (PR-201α, Atago Co., Tokyo, Japan). TA was determined by mixing 5 ml of juice sample with 45 ml of distilled water, titrating the sample to pH 8.1 using 0.1 N NaOH (DL-15 titrator, Mettler-Toledo Ltd., Zurich, Switzerland), and calculating the result as malic acid equivalents. For internal ethylene concentration (IEC) measurement, 1 ml of the gas sampled from the core cavity of each fruit was analyzed using a gas chromatograph (GC-2010, Shimadzu Co., Tokyo, Japan) equipped with an activated column and flame ionization detector. The injector and detector temperatures were set at 200°C, with 90°C oven temperature. The fresh weight loss of individual fruit was recorded before and after storage in both treatments to assess the decrease in weight loss during cold storage. The unit of fresh weight loss rate was expressed as percentage loss of original fruit fresh weight.

Fresh tissue samples were taken from 15 fruit of each treatment during the experiment, cut into small pieces, frozen in liquid nitrogen, and then stored at −80°C until analysis. All analyses were conducted in three biological replicates.

### Extraction and Measurement of the Cell Wall Components

The extraction and measurement of cell wall materials were performed according to the methods of Deng et al. (2005) and Chen et al. (2017a) with slight modifications. Frozen flesh tissue samples (25 g) were added to 200 ml of boiling ethanol, allowed to stand in a boiling water bath for 30 min, and homogenized with a grinder. The resulting slurry was filtered through filter paper (Whatman No. 4), and the residues were washed twice with each of boiling ethanol, chloroform, and acetone, respectively. The crude cell wall extracts were then dried overnight at 30°C. Approximately 500 mg of dried sample was stirred with 70 ml of 50 mM sodium acetate buffer (pH 6.5) at 20°C for 6 h. The supernatant was filtered, and the residue was then washed thrice with 10 ml of the same solution. All supernatants were combined and used as water-soluble pectin (WSP). For CDTA-soluble pectin (CSP), the pellets from WSP were immersed in 50 mM cyclohexane-*trans*-1,2-diaminetetraacetic acid at 20°C for 6 h, and the supernatant was collected, as described above. For Na_2_CO_3_-soluble pectin (NSP), the pellets from CSP were immersed in 50 mM Na_2_CO_3_ containing 100 mM NaBH_4_ at 20°C for 6 h, and the supernatant was collected, as described above. For hemicellulose, the pellet from NSP was immersed in 4% KOH containing 1% NaBH_4_ at 20°C for 6 h, and the supernatant was collected, as described above. The remaining residue was washed with double-distilled deionized water until neutralization, and the mixture was designated for measurement of the cellulose content. The amount of pectin from WSP and NSP was determined by the carbazole method, and calibrated against a galacturonic acid standard curve (Blumenkrantz and Asboe-Hansen, 1973). The content of hemicellulose and cellulose was determined by the anthrone method with a glucose standard curve (D’Amour et al., 1993). Analysis of the cell wall components was carried out on three biological replicates per treatment, and the units (g kg^−1^) were expressed on a fresh weight basis.

### Extraction and Measurement of Activities of Cell Wall-Degrading Enzymes

The cell wall-modifying enzymes were extracted according to the method of Chen et al. (2015) and Chen et al. (2017a). Frozen flesh tissue samples (10 g) were ground with 35 ml of 40 mM sodium acetate buffer (pH 5.2) containing 100 mM NaCl, 2% (v/v) mercaptoethanol, and 5% (w/v) polyvinylpyrrolidone. The homogenate was then centrifuged at 12,000 *g*, 4°C for 30 min, and the supernatant was collected to measure the activities of cell wall-modifying enzymes.

PG and cellulase activities were assayed as described by Deng et al. (2014) and Chen et al. (2017a). A mixture containing 0.5 ml of enzyme extract, 1 ml of 0.5 M sodium acetate (pH 5.5), and 0.5 ml of 1% (w/v) polygalacturonic acid was incubated at 37°C for 1 h and terminated by adding 1.5 ml of 0.63% (w/v) 3,5-dinitrosalicylic acid, heating in a boiling water bath for 5 min, and cooling on ice. The PG concentrations were determined at 540 nm against a blank using a UV-spectrophotometer (UV-1800, Shimadzu Co.) and -galacturonic acid as the standard. One unit of enzyme was defined as the amount of activity released from 1 mmol of galacturonic acid per kilogram per minute on a fresh weight basis.

Cellulase activity was assayed by adding 0.5 ml of enzyme extract and 1.5 ml of 1% (w/v) carboxymethyl cellulose into a test tube and the absorbance measured as per PG mentioned above. One unit of enzyme was defined as the amount of activity released from 1 mmol of -glucose per kilogram per minute on a fresh weight basis.

PME activity was assayed using the method provided by Lin et al. (1989). The mixture of 1 ml of enzyme extract and 4 ml of 1% (w/v) citrus pectin was titrated with 0.01 M NaOH to maintain pH 7.4 while incubating at 37°C for 1 h. One unit of enzyme was defined as the amount of activity released from 1 mmol of NaOH per kilogram per minute on a fresh weight basis.

β-GAL and α-ARF activities were assayed as detailed by Wei et al. (2010) with slight modifications. A mixture containing 0.2 ml of enzyme extract, 0.5 ml of 0.2 M sodium acetate buffer, and 0.2 ml of either 2% (w/v) *p*-nitrophenyl-β-galactopyranoside or *p*-nitrophenyl-α-ARF, respectively, was incubated at 37°C for 1 h and the reaction was stopped by adding 1 ml of 0.1 M Na_2_CO_3_. The absorbance was read at 400 nm against a blank and *p*-nitrophenol was used as the standard. One unit of enzyme was defined as the amount of activity released from 1 mmol of *p*-nitrophenol per kilogram per minute on a fresh weight basis. Analysis of the activities of all cell wall-modifying enzymes was carried out on three biological replicates per treatment.

### Transcriptional Analysis of Genes Encoding Cell Wall-Degrading Enzymes

To verify whether the activities of the cell wall-degrading enzymes were associated with the transcript levels of the candidate genes (*MdPG1*, *MdPME1*, *Mdβ-GAL1*, *Mdβ-GAL2*, and *Mdα-ARF2*), fruit samples (approximately 1 g) were collected from the 1-MCP-treated and untreated (control) fruit stored in a cold room for 1, 3, and 5 months. The samples collected from the untreated fruit that was immediately harvested were also used as a positive control. Total RNA was isolated from the collected samples using an RNAqueous kit (Ambion, Inc., Austin, TX, USA). Using 1 µg of total RNA and an oligo dT20 primer, reverse transcription was performed according to the manufacturer’s instructions (ReverTra Ace-a Toyobo, Osaka, Japan). The transcript levels of the candidate genes (*MdPG1*, *MdPME1*, *Mdβ-GAL1*, *Mdβ-GAL2*, and *Mdα-ARF2*) were determined using a StepOnePlus Real-Time PCR system (Thermo Fisher Scientific, Waltham, MA, USA) (Ai et al., 2016), and gene expression levels were normalized to the actin gene to minimize variation in the cDNA template. The primers and PCR conditions applied to measure the transcript levels are listed in **Supplementary Table S1**. The analysis was repeated three times.

### Statistical Analysis

All statistical analyses were performed with IBM SPSS Statistics 23 software (IBM Corp., Armonk, NY, USA). The independent Student’s *t*-test was used to compare the means between untreated or 1-MCP-treated fruit at ^*^ *P* < 0.05 or ^**^ *P* < 0.01. The results were expressed as means ± standard error.

## Results

### Fruit Quality Attributes

**Figure 1:**
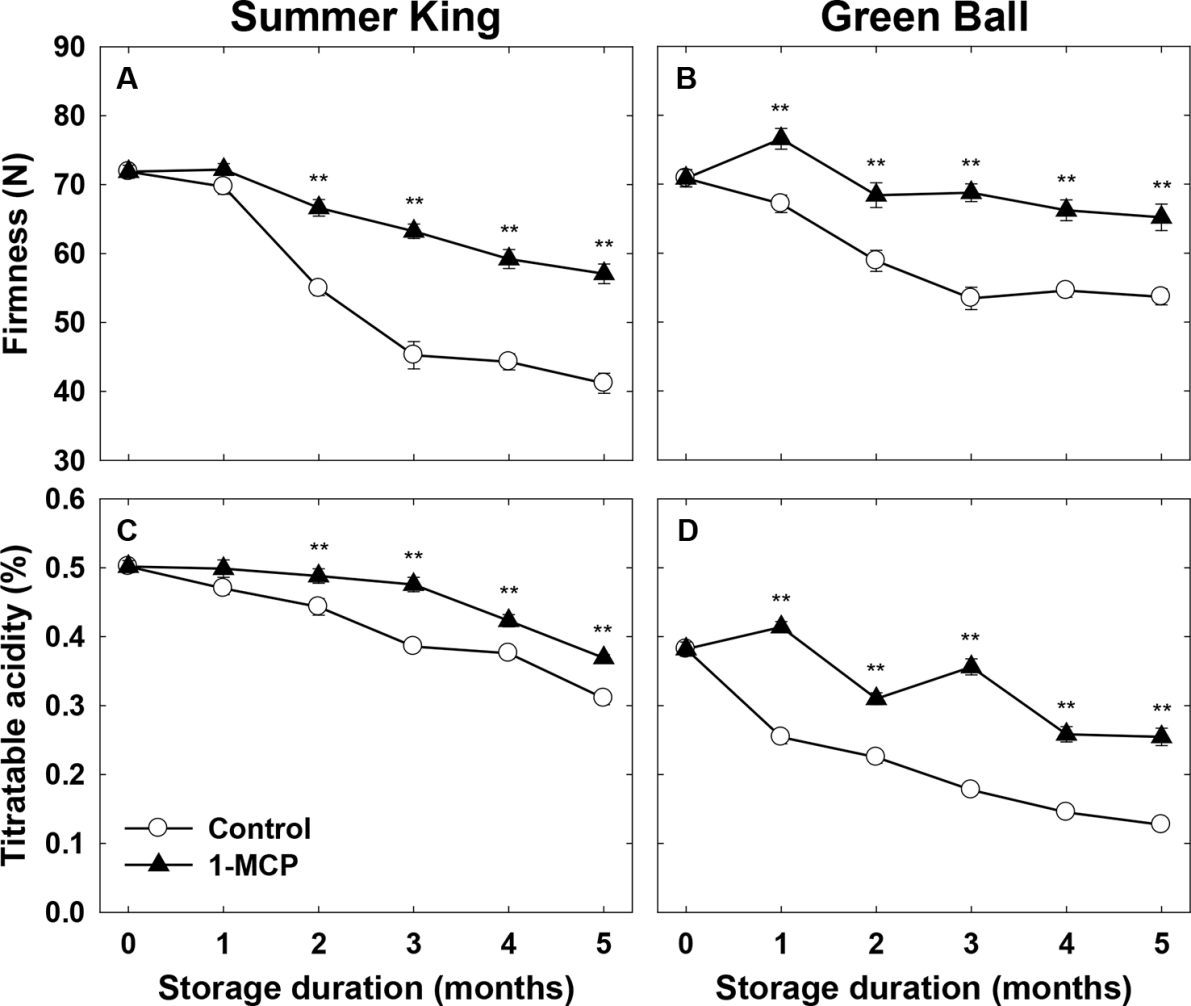
Flesh firmness and titratable acidity of ‘Summer King’ **(A** and **C)** and ‘Green Ball’ **(B** and **D)** apple fruit cultivars (control or treated with 1 μl L^−1^ 1-MCP) at harvest and stored at 0°C for up to 5 months. All values are expressed as means ± standard error (*n* = 15). **P < 0.01.

A significant decline in the flesh firmness was observed in both cultivars throughout cold storage, but this was more significant in the control fruit than those treated with 1-MCP, for both cultivars (**Figures 1A, B**). Similarly, the percentage of TA also significantly declined in both cultivars, most notably in the control fruit than 1-MCP-treated fruit (**Figures 1C, D**). It could be stated that the marked decrease in the flesh vfirmness and TA was directly associated with the increase in storage time, and the control fruit were more affected than those treated with 1-MCP.

**Figure 2:**
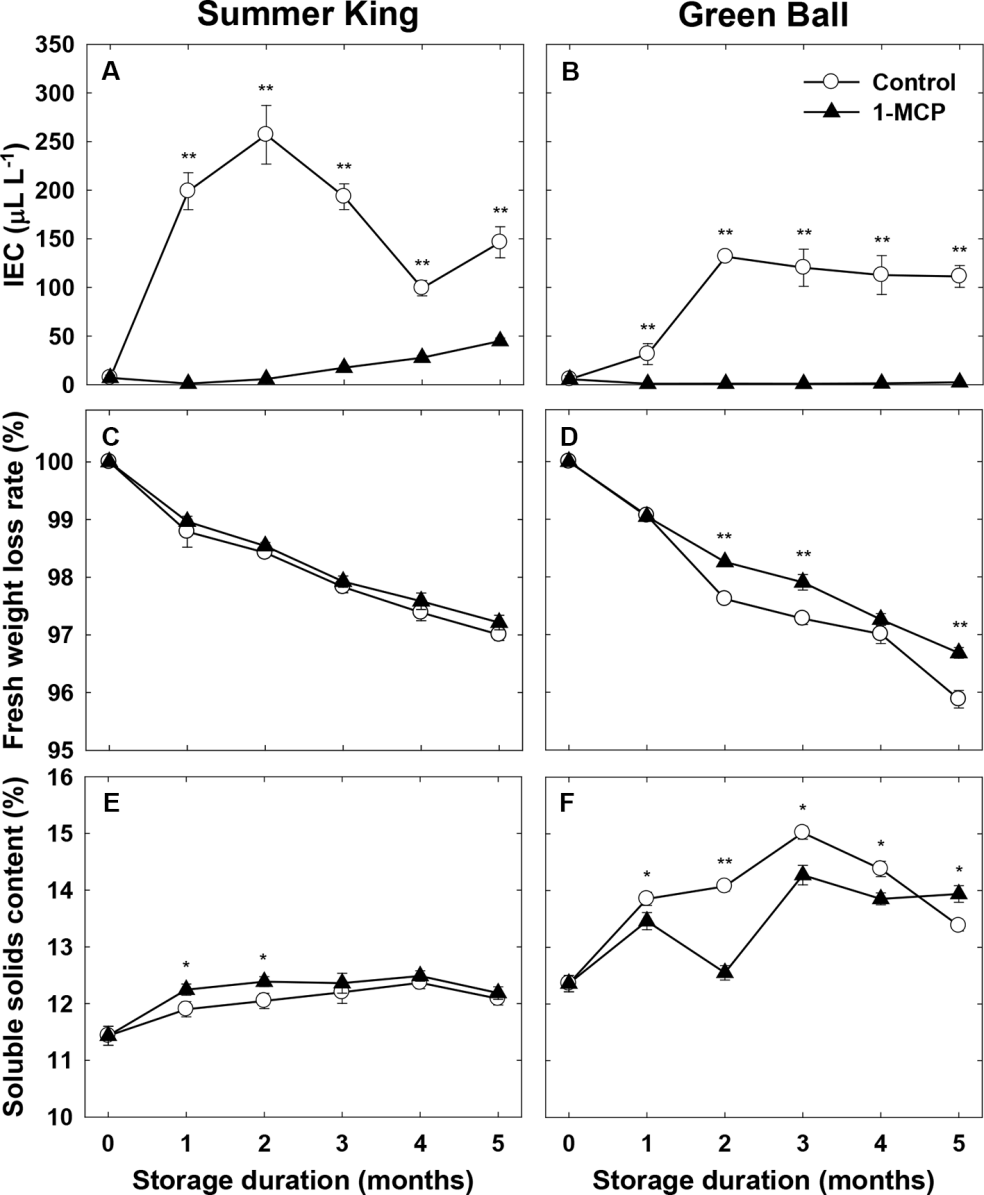
Internal ethylene concentration (IEC), fruit fresh weight loss rate, and soluble solids content of ‘Summer King’ **(A**, **C**, and **E)** and ‘Green Ball’ **(B**, **D**, and **F)** apple fruit cultivars (control or treated with 1 μl L^−1^ 1-MCP) at harvest and stored at 0°C for up to 5 months. All values are expressed as means ± standard error (*n* = 15). ^*^ *P* < 0.05 and ^**^ *P* < 0.01 indicate significant difference between the control and 1-MCP treatment.

In addition, a distinctly lower IEC was induced in 1-MCP-treated fruit compared with the control fruit in both cultivars, which reached an IEC peak at 2 months of storage and declined thereafter (**Figures 2A, B**). Surprisingly, 1-MCP did not significantly affect weight loss for ‘Summer King’ (**Figure 2C**), while it reduced the weight loss of ‘Green Ball’ fruit (**Figure 2D**). The SSC of the two cultivars was also differently affected by 1-MCP treatment during cold storage. Compared with the control fruit, the SSC of 1-MCP-treated fruit remained higher at 1 and 2 months of ‘Summer King’ (**Figure 2E**), while only at 5 months, was the SSC of 1-MCP-treated ‘Green Ball’ greater than that of the control fruit (**Figure 2F**).

### Cell Wall Components

**Figure 3:**
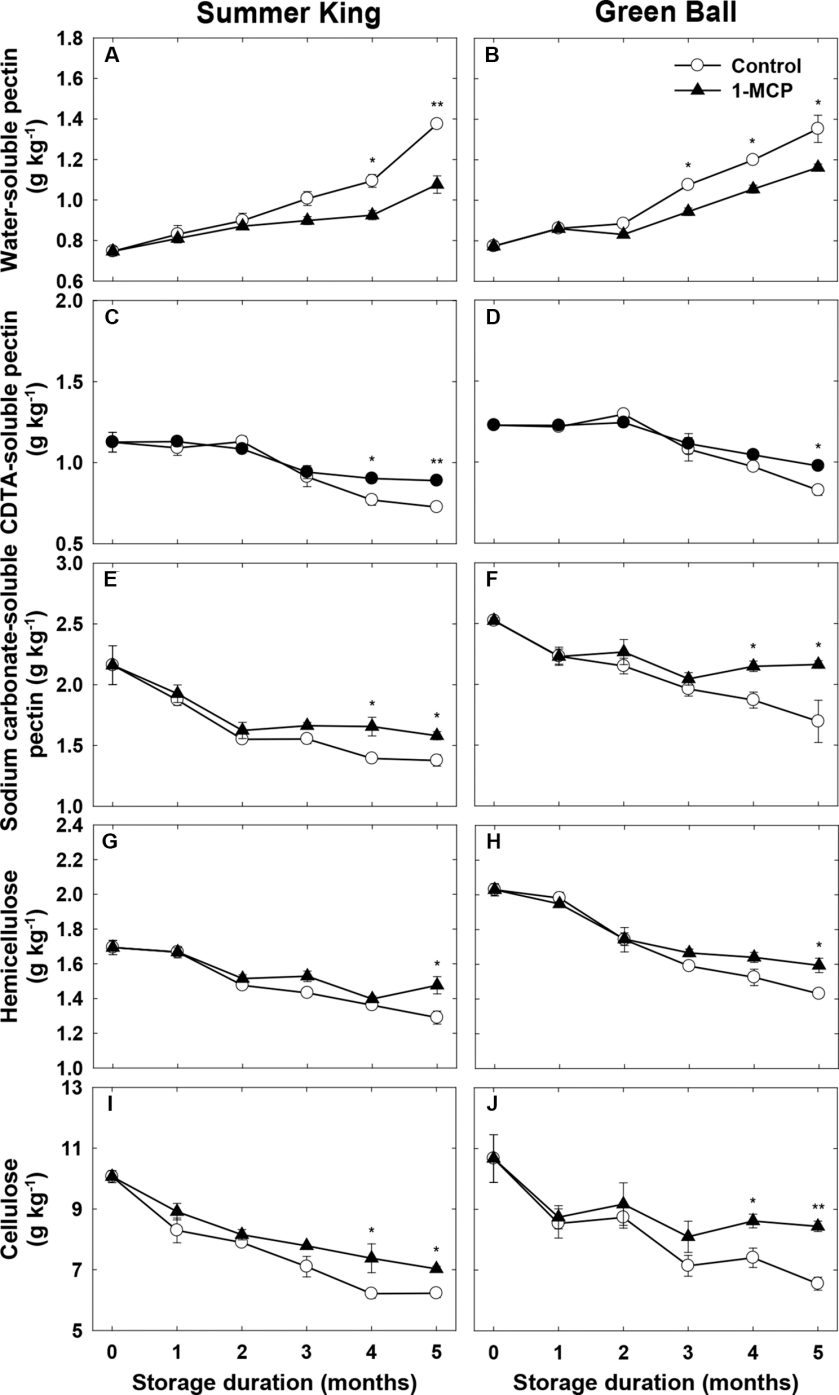
Water-soluble pectin, CDTA-soluble pectin, Na_2_CO_3_-soluble pectin, hemicellulose, and cellulose of ‘Summer King’ **(A**, **C**, **E**, **G**, and **I)** and ‘Green Ball’ **(B**, **D**, **F**, **H**, and **J)** apple fruit cultivars (control or treated with 1 μl L^−1^ 1-MCP) at harvest and stored at 0°C for up to 5 months. All values are expressed as means ± standard error (*n* = 3). *P < 0.05 and **P < 0.01 indicate significant difference between the control and 1-MCP treatment.

An increase in the WSP contents and decrease in the CSP and NSP contents occurred in both cultivars during cold storage (**Figures 3A–F**), in which the WSP contents observed in the control fruit were significantly higher than in the 1-MCP-treated fruit, especially in ‘Summer King’ from 4 months (**Figure 3A**) and ‘Green Ball’ from 3 months (**Figure 3B**). In addition, compared with the control fruit, the 1-MCP treatment also significantly alleviated the storage-induced decrease in CSP and NSP of both cultivars (**Figures 3C–F**). The hemicellulose and cellulose contents tended to decrease in both cultivars as storage time increased but these trends were less dramatic in the 1-MCP-treated fruit, with higher levels of both constituents in these fruit than the control fruit, especially at the later stages (around 4–5 months) of storage (**Figures 3G–J**).

### Cell Wall-Degrading Enzymes

**Figure 4:**
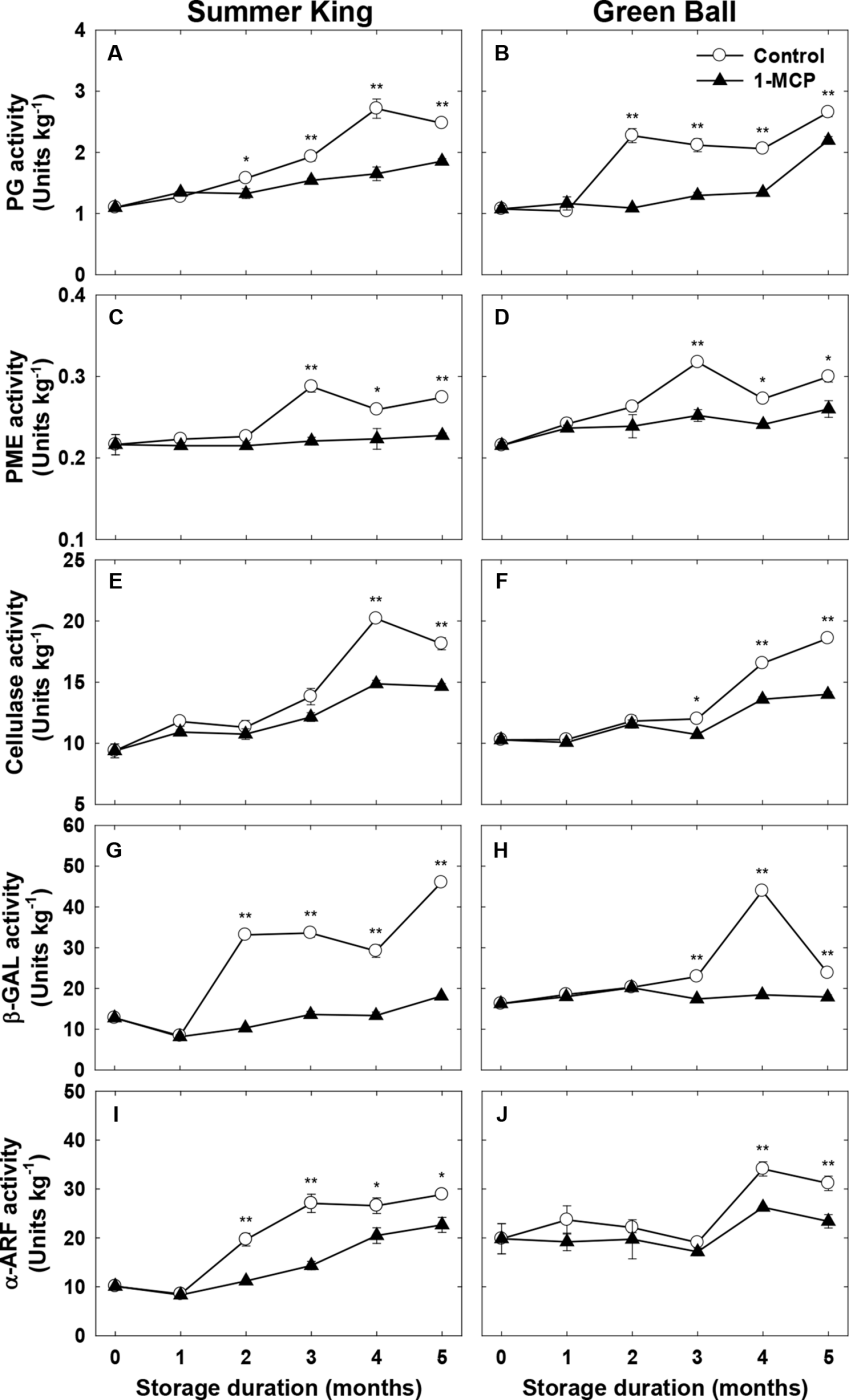
Enzymatic activities of polygalacturonase (PG), pectin methylesterase (PME), cellulose, β-galactosidase (β-GAL), and α-arabinofuranosidase (α-ARF) of ‘Summer King’ **(A**, **C**, **E**, **G**, and **I)** and ‘Green Ball’ **(B**, **D**, **F**, **H**, and **J)** apple fruit cultivars (control or treated with 1 μl L^−1^ 1-MCP) at harvest and stored at 0°C for up to 5 months. All values are expressed as means ± standard error (*n* = 3). *P < 0.05 and **P < 0.01 indicate significant difference between the control and 1-MCP treatment.

To confirm the association between fruit quality and the activities of cell wall-degrading enzymes, the candidate enzymes (PG, PME, cellulase, β-GAL, and α-ARF) that are mainly involved in cell wall degradation were monitored in both cultivars during cold storage. Generally, the activities of the enzymes increased with storage in both cultivars throughout storage (**Figures 4A–J**). Irrespective of the storage duration or enzyme 1-MCP suppressed the enzyme activities, implicating the role of 1-MCP in interfering with the activity of the enzymes, particularly as storage time was prolonged. However, the extent to which 1-MCP affected the enzyme activities varied depending on the type of enzyme (**Figures 4A–J**).

### Pearson’s Correlation Coefficient Analysis

**Figure 5:**
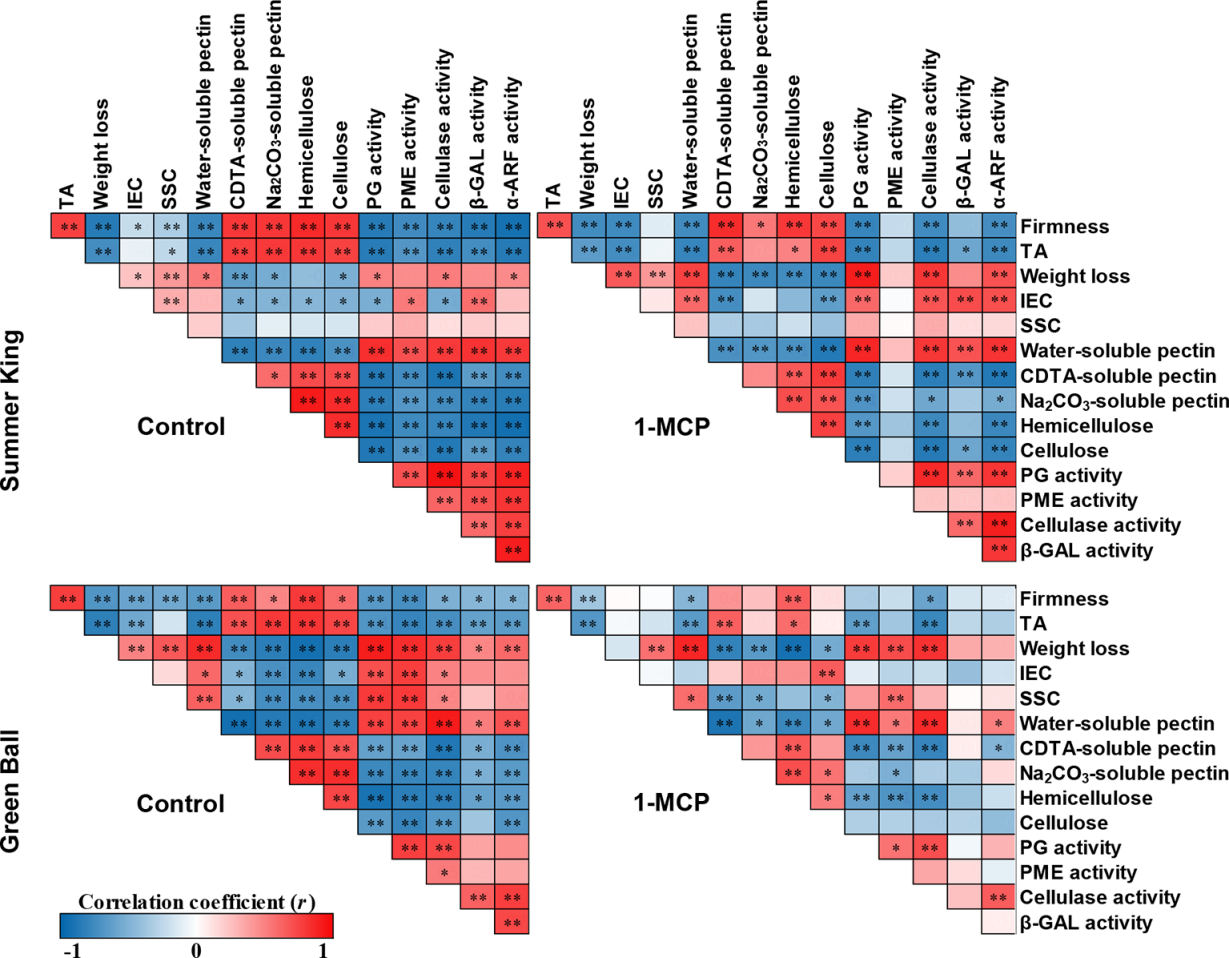
Pearson’s correlation coefficients (*r*) among the analysis of fruit quality attributes, cell wall components, and enzyme activities of control and 1-MCP-treated ‘Summer King’ and ‘Green Ball’ apple cultivars during storage at 0°C for up to 5 months. Red (positive) and blue (negative) correlations between analyses of respective values at *P < 0.05, **P < 0.01.

Pearson’s correlation coefficient test was applied to elucidate the relationship or association between fruit quality attributes and cell wall metabolism of each treatment for the two cultivars during cold storage (**Figure 5**). Among the fruit quality attributes, firmness was positively correlated with TA, and negatively correlated with IEC. However, for 1-MCP-treated ‘Green Ball’ apple, firmness and IEC were not significantly correlated. In addition, firmness was strongly positively correlated with some cell wall components (CSP, NSP, hemicellulose, and cellulose), but negatively correlated with WSP and cell wall-degrading enzymes (PG, PME, cellulase, β-GAL, and α-ARF) for both cultivars. Significant correlations were also greater in the control fruit than 1-MCP-treated fruit of both apple cultivars, especially in 1-MCP-treated ‘Green Ball’ fruit.

### Expression Patterns of the Genes Associated With Cell Wall Degradation

**Figure 6:**
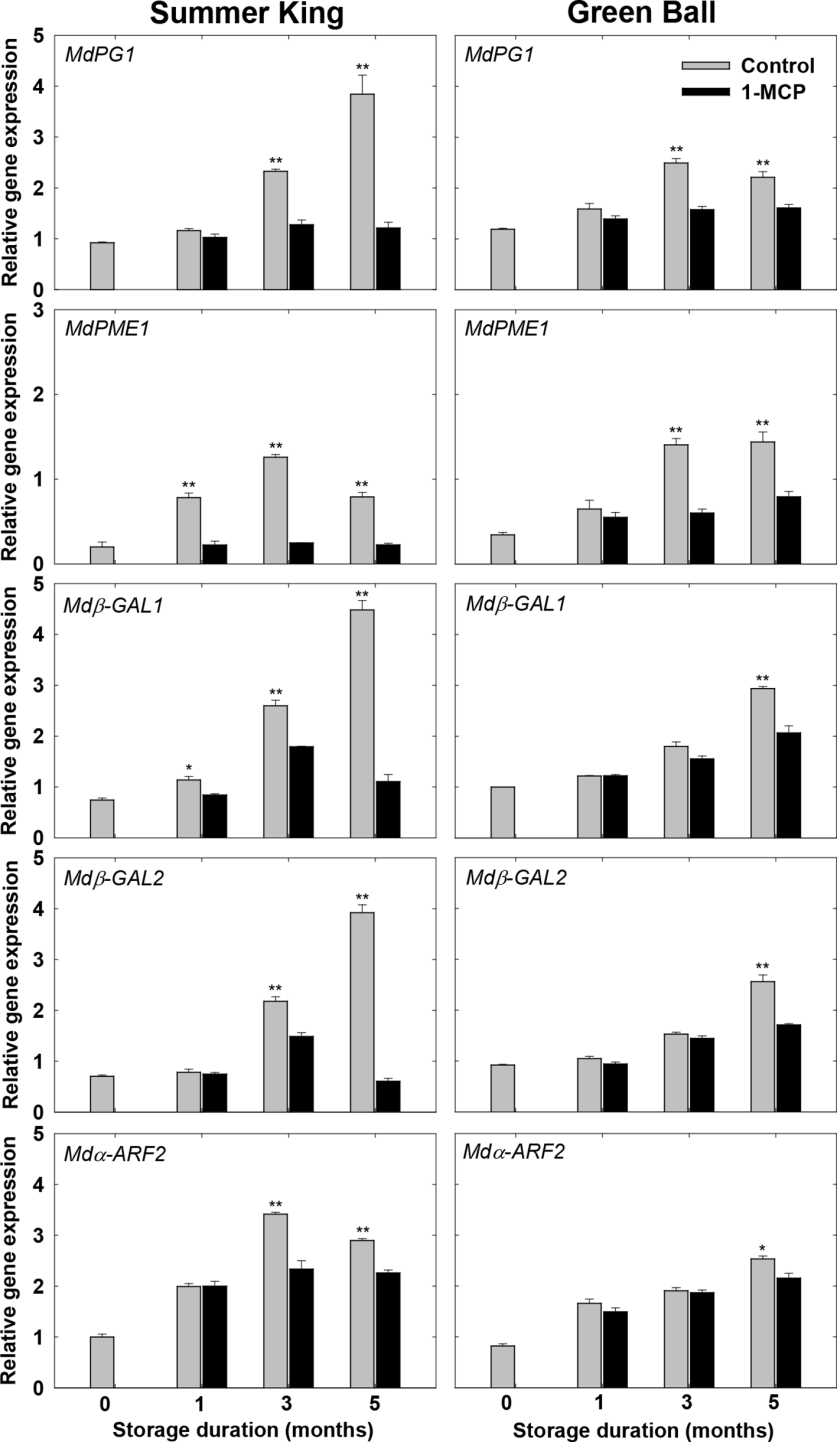
Expression patterns of the genes encoding the cell wall-degrading enzymes in the apple fruit cultivars (control or treated with 1 μl L^−1^ 1-MCP) at harvest and stored at 0°C for up to 5 months. All values are expressed as means ± standard error (*n* = 3). *P < 0.05 and **P < 0.01 indicate significant difference between the control and 1-MCP treatment.

To verify the role of 1-MCP in the regulation of the candidate genes (*MdPG1*, *MdPME1*, *Mdβ-GAL1*, *Mdβ-GAL2*, and *Mdα-ARF2*) associated with cell wall degradation, their transcript levels expressed in 1-MCP-treated and control fruit were detected for both cultivars using qRT-PCR. Differential expression of the genes was observed in the treated and control fruit starting from the early storage times. The transcript levels of the genes tended to be associated with the storage time, increasing with the length of the storage period or increasing until 3 months and then decreasing between 3 and 5 months, especially in the control fruit (**Figure 6**). Overall, the results indicated that 1-MCP was able to alleviate the degradation of cell wall components and the activities of cell wall-degrading enzymes *via* inhibiting the expression levels of the associated genes, in turn, delaying fruit softening and retaining fruit quality during storage.

## Discussion

Maintaining the postharvest quality of apples to meet consumers’ preference is essential for the development of the apple industry. Fruit softening is a major problem of fruit quality, and excessive fruit softening caused by disassembly of cell wall component and degradation of cell walls during storage leads to economic loss. To delay the excessive fruit softening, 1-MCP has been exploited in postharvest biology research and its effect on delaying fruit softening during long storage periods *via* the inhibition of the disassembly of cell wall components and the activities of cell wall-degrading enzymes have also been reported (Watkins, 2006; Watkins, 2008; Kim et al., 2018; Lu et al., 2018). However, its effect on fruit softening and quality varies, depending on the type of cultivar studied (Watkins et al., 2000; Delong et al., 2004). Hence, it was interesting to investigate whether 1-MCP could delay fruit softening of the newly released apple cultivars ‘Summer King’ and ‘Green Ball’. In the present study, we investigated the role of 1-MCP in fruit softening of the two apple cultivars by determining the fruit quality attributes, cell wall components, and the activities of cell wall-degrading enzymes and the expression levels of their associated genes.

Fruit firmness and TA determine the quality of fruit and so delaying the loss of these attributes during storage is economically important. In this study, 1-MCP diminished the loss of fruit firmness and TA in both cultivars, as compared with the control fruit. These results are consistent with those of previous studies reporting the positive effect of 1-MCP on the maintenance of flesh firmness and TA under air or controlled atmosphere storage (Bai et al., 2005; Watkins, 2008; Kim et al., 2018; Yoo et al., 2018). The trend of firmness and TA to decrease was similar between these attributes, with a high positive correlation with loss of fruit quality during storage.

In addition, 1-MCP significantly suppressed the IEC in both cultivars, which is mainly involved in fruit ripening, but the IEC observed in ‘Summer King’ apples was higher than that of ‘Green Ball’ apples. This discrepancy could be due to the difference between the concentration of IEC induced by the two different cultivars (Jung and Watkins, 2014; Yoo et al., 2016). Yoo et al. (2016) reported that low IEC is linked to less reduction of firmness, as observed in this study.

However, the effect of 1-MCP on weight loss was influenced by cultivar, because different to that seen in ‘Green Ball’, 1-MCP was not able to delay weight loss in ‘Summer King’ when compared with the control fruit. Kim et al. (2018) observed that the SSC increase is associated with an increase in weight loss. Similarly, this study confirmed the connection between SSC and weight loss in the treated and control fruit for both cultivars during storage. Watkins et al. (2000) and DeEll et al. (2002) suggested that 1-MCP treatment can result in lower, higher or similar SSC compared with control fruit during storage, depending on cultivar, which was also consistent with our results. The decrease in firmness had a strong correlation with the increase in weight loss, especially in ‘Green Ball’.

Fruit softening is primarily caused by the degradation of cell wall components, including pectin, hemicellulose, and cellulose (Brummell and Harpster, 2001; Deng et al., 2005; Vicente et al., 2007). Pectin is one of the main constituents of the primary cell wall and middle lamella, and the further breakdown of pectin leads to the disassembly of the hemicellulose and cellulose network of the cell wall structure, resulting in softening (Wu et al., 2011; Chen et al., 2017a; Chen et al., 2017b). In addition, the involvement of the WSP, NSP, and hemicellulose contents in the degradation process of the cell wall components during storage of different apple cultivars has been demonstrated (Oritz et al., 2011a; Oritz et al., 2011b; Gwanpua et al., 2014; Gwanpua et al., 2017). Our findings also verified the correlation between the firmness and cell wall components (pectin, hemicellulose, cellulose, WSP, CSP, and NSP), whereas 1-MCP treatment delayed the degradation of the cell wall components in both cultivars.

The disassembly of cell wall components occurs primarily as a result of the activities of cell wall-degrading enzymes (PG, PME, β-GAL, and α-ARF) (Brummell et al., 2004; Wei et al., 2010; Gwanpua et al., 2014; Gwanpua et al., 2016). The degradation of pectic substances is closely related to the action of PG and PME activities, which plays a significant role in the modifications of fruit tissues during ripening (Brummell and Harpster, 2001; Brummell et al., 2004; Rodoni et al., 2010; Wei et al., 2010). In this study, a strongly positive correlation between PG and PME activities was found in the control fruit of both cultivars. Furthermore, these activities were correlated with firmness loss, as previously reported in ‘Golden Delicious’ and ‘Fuji’ apples (Wei et al., 2010). Moreover, the increasing storage time showed an increased degradation of enzyme activities, which was most evident in the control fruit. Therefore, this study suggested that 1-MCP delayed the fruit softening process due to depolymerization and dissolution of pectin in both cultivars.

Cellulase is associated with the disassembly of cellulose and xyloglucan in the cell walls of fruit (Bu et al., 2013; Chen et al., 2017a; Chen et al., 2017b; Shi et al., 2019). β-GAL and α-ARF are responsible for the loss of cell wall integrity that leads to softening (Payasi et al., 2009; Wei et al., 2010). The role of β-GAL activity in initial cell wall processes during apple fruit softening has also been documented (Wei et al., 2010; Gwanpua et al., 2016). However, the release of arabinosyl residues from pectin was found to be relatively low during ripening and increased dramatically at the over-ripening stage (Goulao et al., 2007). In this study, an increase in the enzyme activities was observed during storage, with higher activities in the control fruit than 1-MCP-treated fruit, and this increase was closely associated with the IEC and firmness, prompting apple fruit softening. The expression levels of the genes encoding the cell wall-degrading enzymes further supported this association between the activities of the enzymes and apple fruit softening because their expression levels were significantly higher in the control fruit than treated fruit, regardless of the storage duration. The strong suppression of *MdPG* in apple by 1-MCP has been reported (Wakasa et al., 2006; Mann et al., 2008). Moreover, Wei et al. (2010) claimed that 1-MCP dramatically postponed fruit softening by inhibiting β-GAL activity and the expression level of β-GAL In this study, the expression levels of *Mdβ-GAL1* and *Mdβ-GAL2* continuously increased till the end of storage (5 months), with a higher expression in the control fruit than treated fruit, indicating the inhibitory role of 1-MCP on expression of the genes. Likewise, Gwanpua et al. (2016) affirmed the enhanced expression of both *Mdβ-GAL1* and *Mdβ-GAL2* during ripening and an inhibitory role of 1-MCP in the expression of the genes. The involvement of *Mdα-ARF* and *MdPE1* in the cell wall degradation and fruit softening of apple was also reported (Gwanpua et al., 2016). Taken together, the exploitation of 1-MCP in the newly released apple cultivars allowed delaying their fruit softening *via* curtailing the cell wall decomposition, and suppressing the cell wall degradation-related genes and their associated enzyme activities, which are mainly involved in the sustainable maintenance of the postharvest quality of the fruit.

## Conclusion

This study has demonstrated that 1-MCP treatment delayed the fruit softening of ‘Green Ball’ and ‘Summer King’ apples cultivars. The softening of apple fruit was related to the disassembly of cell wall components that resulted from the actions of cell wall-degrading enzymes. More rapid loss of firmness was correlated with a faster degradation of cell wall components and higher activities of enzymes that are directly linked to fruit softening. The escalated loss of fruit quality and cell wall degradation is related to the increase in storage time. This study suggested that the storage of the new cultivars with 1-MCP treatment would be an effective technology for the apple industry, due to its ability to notably delay fruit softening during long storage periods, and these differential responses encountered in this study can be used to further explore physiological processes associated with fruit ripening.

## Data Availability Statement

All datasets for this study are included in the article/supplementary material.

## Author Contributions

NW mainly conducted the experiment and JY assisted the experiment. S-IK and CW advised the experiment. NW wrote the manuscript. NW and JY revised the manuscript. I-KK supervised the project.

## Funding

This work was supported by the 2017 research fund of the Rural Development Administration, Republic of Korea (grant number PJ012455022017).

## Conflict of Interest

The authors declare that the research was conducted in the absence of any commercial or financial relationships that could be construed as a potential conflict of interest.
