# Supplementary material for: Characterization of Fruit Quality Attributes and Cell Wall Metabolism in 1-Methylcyclopropene (1-MCP)-Treated ‘Summer King’ and ‘Green Ball’ Apples During Cold Storage
Source: Front Plant Sci. 2019 Nov 21;10:1513. doi: 10.3389/fpls.2019.01513 (PMC6882424; doi:10.3389/fpls.2019.01513)
Supplement: Supplementary file 1 [file Table_1.docx]

# Supplementary Data

**Supplementary Table 1:** Primer sequences used in the gene expression studies of softening-related candidate genes.

| **Gene** | **Accession** | **F-primer** | **R-primer** |
| --- | --- | --- | --- |
| Mdα-AF2 | XM_029090787.1 | CAGTCCTAATTCTACATGGGGTTC | CCCAATAGCAACATATCTCAAGTC |
| Mdβ-GAL1 | XM_029094068.1 | AGACACTGGAGTTCCATGGATTAT | ACCGAATTCTGTATACCAACCAGT |
| Mdβ-GAL2 | NM_001293826.1 | CAATTCACTATCCCAGAAGCACTC | CTTGACCAAATCATATCTGTCCTC |
| MdPG1 | [NM_001293928.1](https://www.ncbi.nlm.nih.gov/nucleotide/NM_001293928.1?report=genbank&log$=nuclalign&blast_rank=8&RID=GPP4F8EV015) | CCCAGACAAACCTTAATCACTTCT | GAAGCACTCGTTCCTCTTATGTT |
| MdPME1 | XM_008349560.3 | CTCTGTTGCTAGTGGTGGGTACTT | ATCCATCTCCTACGCTGTTATTTC |
| MdActin | CN938023 | TGACCGAATGAGCAAGGAAATTACT | TACTCAGCTTTGGCAATCCACATC |

**PCR condition**

The cycling conditions were as follows: denaturation step at 95 _C for 10 min, followed by 45 cycles of denaturation at 95 _C for 20 s, annealing at 63 _C for 20 s, and extension at 72 _C for 20 s.
